# Malaria 2050: from science to strategy; from evidence to eradication

**DOI:** 10.1186/1475-2875-13-S1-O9

**Published:** 2014-09-22

**Authors:** Richard Feachem

**Affiliations:** 1 The Global Health Group, University of California, San Francisco (UCSF), USA

## 

The malaria community has moved rapidly from acceptance of the possibility of elimination in some countries to an active discussion about eventual human malaria eradication, perhaps by 2050. If this ambitious goal is to be achieved, several things are necessary.

**First**, we must have a clear overall strategy for the achievement of malaria eradication. This involves three equally important elements:

• Aggressive control in the heartland to bring malaria burden from high to low.

• Shrinking the malaria map by eliminating transmission in countries at the endemic margins.

• Research to bring forward new drugs, vaccines, insecticides and other tools and technologies.

**Second**, we must have a clear roadmap leading to eradication in 2050. If all countries have eliminated by 2050, what must the 2040 map look like? What must the 2030 map look like? And so on. We must set ambitious but achievable five-yearly targets. These must be accompanied by research timelines for the discovery of new and better tools and technologies.

**Third**, we must aggressively use today’s tools today and be ready to adopt and rapidly scale-up tomorrow’s tools for tomorrow.

**Fourth**, we must work to strengthen both national programs and regional collaborations. Two significant regional collaborations, APMEN and E8, provide models.

**Fifth**, we must overcome specific technical challenges including artemisinin-resistant *P. falciparum*, *Plasmodium knowlesi*, cross-border malaria movement, and the systemically weak management that is found in most National Malaria Control Programs.

**Sixth**, we must ensure adequate finance, not only to reach elimination, but to prevent reintroduction. The possibility of declining budgets in the face of declining malaria, and no budgets in the face of no malaria, is ever present. Sri Lanka in the 1960s will repeat itself if we are not proactive. We vaccinate against measles where there is no measles. We need to invest in malaria control where there is no malaria.

Finally, we must continue to transition from a science-led malaria endeavor to a science-supported malaria endeavor. Scientists will always focus on what we do not know and on the lack of evidence to support aggressive action. Leadership needs to come from those who are experienced in bold action based on sufficient evidence, to create a more ambitious trajectory toward eradication. And this leadership needs to be supported by the best scientists and the most vigorous development of new products and tools. Linked to this culture of action, must be an emphasis on operational research and learning while doing.

With these and other commitments and efforts, we can collectively embrace the vision of a planet with no human malaria by 2050.

